# Chronic intestinal inflammation drives colorectal tumor formation triggered by dietary heme iron in vivo

**DOI:** 10.1007/s00204-021-03064-6

**Published:** 2021-05-12

**Authors:** Nina Seiwert, Janine Adam, Pablo Steinberg, Stefan Wirtz, Tanja Schwerdtle, Petra Adams-Quack, Nadine Hövelmeyer, Bernd Kaina, Sebastian Foersch, Jörg Fahrer

**Affiliations:** 1 Institute of Toxicology, University Medical Center Mainz, 55131 Mainz, Germany; 2 Rudolf Buchheim Institute of Pharmacology, Justus Liebig University Giessen, 35392 Giessen, Germany; 3 Division of Food Chemistry and Toxicology, Department of Chemistry, Technical University of Kaiserslautern, Erwin-Schroedinger-Str. 52, 67663 Kaiserslautern, Germany; 4 Institute for Food Toxicology and Analytical Chemistry, University of Veterinary Medicine, 30173 Hannover, Germany; 5 Max-Rubner-Institute, 76131, Karlsruhe, Germany; 6 Medical Department 1, University Medical Center, 91054 Erlangen, Germany; 7 Department of Food Chemistry, Institute of Nutritional Science, University of Potsdam, 14558 Nuthetal, Germany; 8 TraceAge—DFG Research Unit on Interactions of Essential Trace Elements in Healthy and Diseased Elderly (FOR 2558), Berlin-Potsdam-Jena , Germany; 9 Institute for Molecular Medicine, University Medical Center Mainz, 55131 Mainz, Germany; 10 Institute of Pathology, University Medical Center Mainz, 55131 Mainz, Germany

**Keywords:** Dietary heme, Intestinal inflammation, Colorectal tumorigenesis, DNA damage, Mouse models

## Abstract

The consumption of red meat is associated with an increased risk for colorectal cancer (CRC). Multiple lines of evidence suggest that heme iron as abundant constituent of red meat is responsible for its carcinogenic potential. However, the underlying mechanisms are not fully understood and particularly the role of intestinal inflammation has not been investigated. To address this important issue, we analyzed the impact of heme iron (0.25 µmol/g diet) on the intestinal microbiota, gut inflammation and colorectal tumor formation in mice. An iron-balanced diet with ferric citrate (0.25 µmol/g diet) was used as reference. 16S rRNA sequencing revealed that dietary heme reduced α-diversity and caused a persistent intestinal dysbiosis, with a continuous increase in gram-negative *Proteobacteria*. This was linked to chronic gut inflammation and hyperproliferation of the intestinal epithelium as attested by mini-endoscopy, histopathology and immunohistochemistry. Dietary heme triggered the infiltration of myeloid cells into colorectal mucosa with an increased level of COX-2 positive cells. Furthermore, flow cytometry-based phenotyping demonstrated an increased number of T cells and B cells in the *lamina propria* following heme intake, while γδ-T cells were reduced in the intraepithelial compartment. Dietary heme iron catalyzed formation of fecal *N*-nitroso compounds and was genotoxic in intestinal epithelial cells, yet suppressed intestinal apoptosis as evidenced by confocal microscopy and western blot analysis. Finally, a chemically induced CRC mouse model showed persistent intestinal dysbiosis, chronic gut inflammation and increased colorectal tumorigenesis following heme iron intake. Altogether, this study unveiled intestinal inflammation as important driver in heme iron-associated colorectal carcinogenesis.

**Supplementary Information:**

The online version contains supplementary material available at 10.1007/s00204-021-03064-6.

## Introduction

More than 1.8 million people worldwide are diagnosed with colorectal cancer (CRC) annually, thus ranking CRC as one of the major tumor entities (Keum and Giovannucci 2019). CRC incidence and death rates have been continuously rising in adults younger than 50 years both in the US and Europe (Siegel et al. 2020; Vuik et al. 2019). Colorectal carcinogenesis is causally linked to various factors, including genetic predisposition, inflammatory bowel disease (IBD), lifestyle factors and nutrition (Kuipers et al. 2015; Murphy et al. 2019). The consumption of red and processed meat correlates with an increased risk to develop CRC as revealed by epidemiological studies (Chao et al. 2005; Mehta et al. 2020). Several years ago, the International Agency for Research on Cancer classified the intake of processed meat as carcinogenic in humans, while red meat consumption was assessed as probably carcinogenic due to limited mechanistic evidence (Bouvard et al. 2015). The carcinogenic potential of red meat is most likely attributable to its constituent heme (Seiwert et al. 2020a), which is the prosthetic group of both hemoglobin and myoglobin and occurs abundantly in red meat (Lombardi‐Boccia et al. 2002). Furthermore, viral bovine meat and milk factors as well as a non-human sialic acid found in red meat may also be involved in CRC development (Samraj et al. 2015; Zur Hausen et al. 2019).

The mechanisms underlying heme iron-triggered colorectal carcinogenesis are not fully understood, but include increased formation of genotoxic agents such as *N*-nitroso compounds (NOC) and reactive oxygen species (ROS), aberrant proliferation of the intestinal epithelium due to altered WNT signaling as well as alterations in the intestinal microbiota (Bastide et al. 2015; IJssennagger et al. 2012b; Martin et al. 2019; Seiwert et al. 2020b). The latter was shown to go along with a reduced mucus barrier (Ijssennagger et al. 2015), thereby likely increasing the exposure of the intestinal epithelium to luminal bacteria. It is unclear, however, whether dietary heme affects the intestinal immune system and can trigger intestinal inflammation. A short-term study over a period of 14 days in mice fed with a diet supplemented with heme iron did not observe an immune response or activation of inflammatory pathways in gut epithelium (IJssennagger et al. 2012a). In contrast, two other studies revealed that dietary heme aggravates chemically induced colitis in rodents (Constante et al. 2017; Schepens et al. 2011). Both chronic intestinal inflammation as observed in patients with inflammatory bowel disease and inflammatory processes in the tumor microenvironment found in sporadic CRC are known to promote colorectal carcinogenesis (Lasry et al. 2016). Interestingly, heme administered by tail vein injection at high concentration causes inflammation in the liver and other organs (Wagener et al. 2001).

We hypothesized that chronic dietary uptake of heme iron may trigger persistent changes in the gut microbiota as well as intestinal inflammation, which in turn would promote CRC formation. To address these important issues and elucidate the mechanisms underlying heme-triggered carcinogenesis, we first analyzed the impact of dietary heme iron on the intestinal microbiota in C57BL/6 J mice as compared to a control diet with iron citrate in a long-term study. Gut inflammation was monitored by non-invasive mini-endoscopy and colorectal tissue was collected for histopathological analysis. The infiltration of immune cells and markers of inflammation were assessed by immunohistochemistry, while effects on distinct T cell populations in both the *lamina propria* and the intraepithelial compartment were analyzed by flow cytometry. Furthermore, the formation of fecal NOC, DNA damage in the intestinal epithelium and apoptotic cell death was studied. Finally, colorectal tumor formation was investigated by mini-endoscopy using the colonotropic alkylating agent azoxymethane (AOM) as tumor initiator followed by a long-term diet with heme iron or iron citrate as control.

## Materials and methods

### Animal experiments, feeding study and mini-endoscopy

**Fig. 1:**
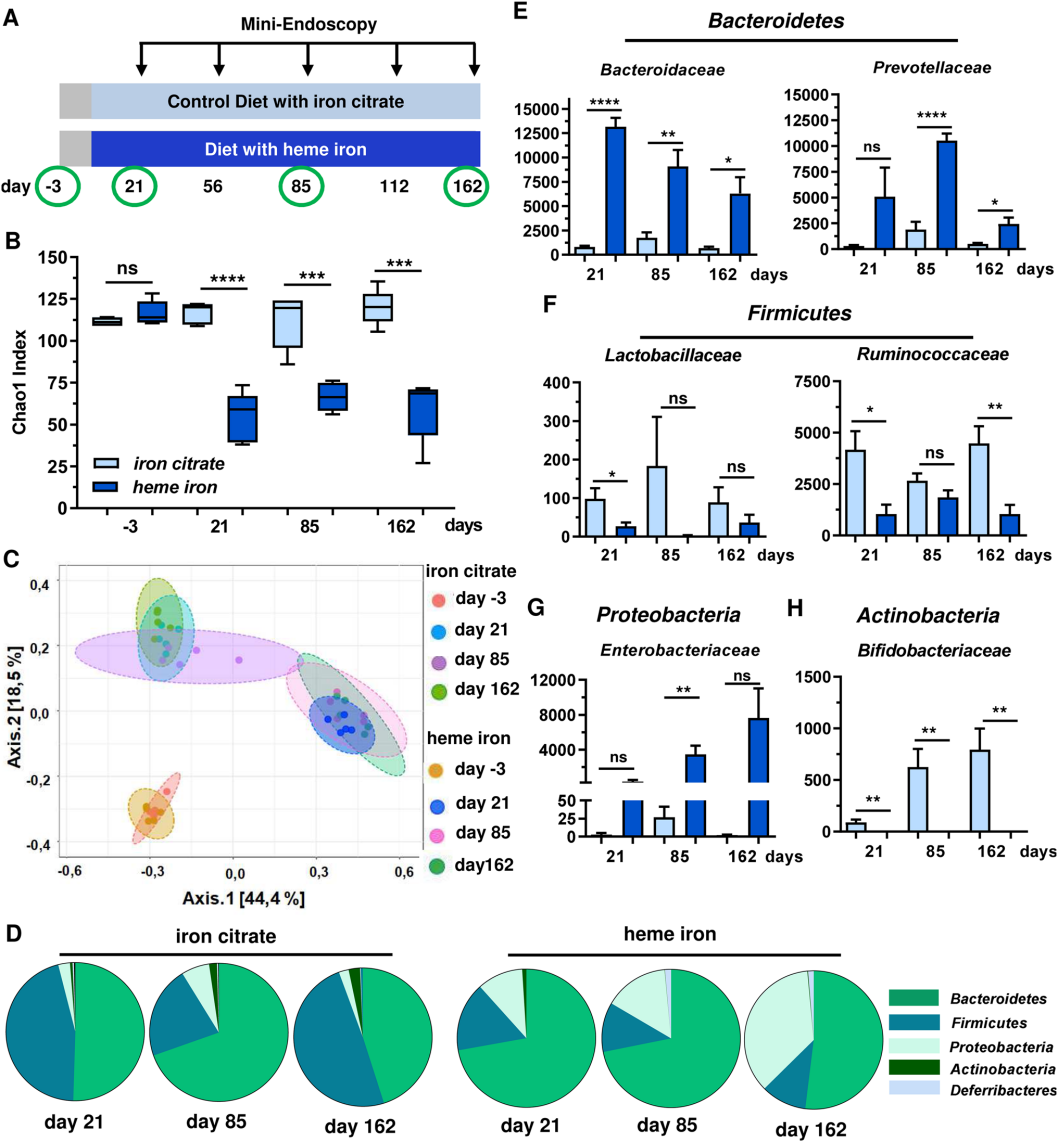
Dietary heme iron causes a persistent intestinal dysbiosis with reduced α-diversity. **a** Experimental setup. C57BL/6 J mice received an initial *i.p.* injection of PBS. After 72 h, the mice were set on a diet supplemented with heme iron or a control diet with iron citrate for up to 162 days. Mini-endoscopy was performed at indicated time points. Fecal samples for microbiome analysis were freshly collected at the indicated time points (green circles). **b** α-diversity of the intestinal microbiome shown as Chao1 Index. Data are depicted as median with minimum to maximum (*n* = 5 mice per group). Ns: *p* > 0.05; ***p < 0.001; ****p < 0.0001. **c** β-diversity of the intestinal microbiome shown as Bray Curtis dissimilarity. Shown are single data points. **d** Mean distribution of selected intestinal bacterial phyla in mice fed for 21, 85 and 162 days (*n* = 5 per group). **e**–**h** Relative abundance of *Bacteroidetes* (**e**), *Firmicutes* (**f**), Proteobacteria (**g**), and *Actinobacteria* (**h**) after 21, 85 and 162 days. Shown are mean + SEM (*n* = 5). Ns: *p* > 0.05; *p < 0.05; **p < 0.01; ***p < 0.001; ****p < 0.0001

Eight- to 16-week-old sex-matched C57BL/6 J mice were obtained from the in-house animal breeding facility at University Medical Center (Mainz, Germany) and randomized into two treatment groups. For microbiome analysis, only female mice purchased at Envigo (Indianapolis, USA) were used (further details in the section below). For the feeding study, animals received iron-balanced experimental diets (Supplementary Table 1) ad libitum for the indicated time. Heme iron was supplemented as hemin (Sigma Aldrich, Schnellendorf, Germany) at a final dose of 0.25 µmol/g diet. An iron-balanced diet supplemented with ferric citrate (0.25 µmol/g diet) served as control. The experimental diets were produced by Altromin (Lage, Germany). To analyze the tumor promoting properties of heme iron, a single initial *i.p.* injection with the colonotropic alkylating agent AOM diluted in PBS (15 mg/kg body weight) was administered. 72 h after the AOM administration, the animals received the experimental diets for up to 162 days. Intestinal inflammation was monitored regularly using a high-resolution mini-endoscopy system (Karl Storz, Tuttlingen, Germany) at different time points (see Fig. 1a). The inflammation score was assessed as murine endoscopic index of colitis severity (MEICS) according to our previous study (Dörsam et al. 2018). MEICS is based on five parameters: thickening of the colon wall, changes of the vascular pattern, granularity of the colonic mucosal surface, presence of fibrin and stool consistency (Becker et al. 2006). Colorectal tumors were scored with regard to their number and size as described (Fahrer et al. 2015). The evaluations were done in a not-blinded fashion.

### Preparation of protein lysates from colorectal tissue

Tissue lysates were prepared as reported and protein content was determined using the Bradford Assay (Fahrer et al. 2015).

### SDS-PAGE and immunoblotting via Western blot

Samples were separated by SDS-PAGE followed by the transfer onto a nitrocellulose membrane (Perkin Elmer, Rodgau, Germany) via wet blot technique (BioRad, Munich, Germany) as described (Mimmler et al. 2016). Primary antibodies and horseradish-peroxidase conjugated secondary antibodies (Supplementary Table 2) were used for chemoluminescence detection with Western Lightning® Plus-ECL (Perkin Elmer, Rodgau, Germany).

### Tissue collection, processing and histopathological analysis

Tissue biopsies from distal colon were harvested as indicated and either snap frozen in liquid nitrogen or fixed in neutral buffered formaldehyde solution (Roti-Histofix, Carl Roth, Karlsruhe, Germany) as mentioned previously (Dörsam et al. 2018). For histological analysis, fixed tissue was embedded in paraffin and cut into 5 µm-thick sections using a microtome (Carl Zeiss, Oberkochen, Germany) followed by hematoxylin and eosin (H&E) staining (Dörsam et al. 2018). Finally, stained sections were scanned with a Zeiss Axiovert 35 microscope equipped with an Olympus Colorview I camera. Using NDP.view2-Software images were processed and histopathological parameters like crypt length was determined.

### Immunohistochemistry and confocal microscopy

Paraffin-embedded sections of formaldehyde-fixed colon tissue were processed, stained with primary antibodies followed by fluorophore-conjugated secondary antibodies (Supplementary Table 3) and analyzed by confocal microscopy as reported (Fahrer et al. 2015). Apoptotic cells were detected in situ with TUNEL assay (Roche Diagnostics, Mannheim, Germany) as published (Fahrer et al. 2015).

### ICP–MS analysis to determine total iron levels in colorectal tissue

Total iron levels in snap-frozen colorectal tissue from mice fed with heme iron or ferric citrate were determined by ICP–MS essentially as described (Seiwert et al. 2020b).

### Isolation of genomic bacterial DNA from murine stool for microbiome analysis

Female C57BL/6 J mice (7–8 weeks old) were obtained from Envigo (Indianapolis, USA), randomly grouped in two treatment groups with 2–3 mice per cage and acclimatized for 1 week. One group received a diet supplemented with hemin (0.25 µmol/g diet), whereas the other group received an iron-balanced diet supplemented with ferric citrate (0.25 µmol/g diet) as control (each group *n* = 5). For experimental design and sample schedule, see Fig. 1a. Fresh stool samples were collected as indicated, snap frozen immediately and stored at − 80 °C. Isolation of bacterial genomic DNA was performed using PSP® Stool DNA Plus Kit (Stratec Molecular GmbH, Berlin, Germany). Finally, the DNA content and purity were determined with a NanoDrop 2000 (Thermo Scientific, Braunschweig, Germany) and verified with a Qubit Fluorometer using a dsDNA HS Assay Kit (Thermo Scientific, Braunschweig, Germany). For the analysis of intestinal microbial changes in AOM-initiated animals, female C57BL/6 mice (7–8 weeks old; Envigo) received an initial i.p. injection of AOM (15 mg/kg bw) at day − 3. After 72 h, the mice were set on a diet supplemented with hemin or a control diet with iron citrate for up to 162 days (*n* = 5 per group; 2–3 animals per cage). Fecal samples for microbiome analysis were freshly collected at the indicated time points (see Figure S6A for experimental setup) and processed as described above.

### Next generation sequencing (NGS)

To amplify V3 and V4 regions of 16S rRNA genes, 10 ng bacterial template DNA was used. Degenerative region-specific and barcode-containing primers and Illumina Flow Cell adapter sequences as well as the NEBNext® Ultra™ II Q5® Mastermix (NEB Inc., Massachusetts, USA) were used (Fadrosh et al. 2014). Amplicons were purified with Agencourt AMPure XP beads (Beckman Coulter Life Science, Krefeld, Germany), normalized and run on the MiSeq System (Illumina, San Diego, USA) using a 600-cycle end-paired kit and the standard Illumina HP10 and HP11 sequencing primers. The resulting fastq files were evaluated bioinformatically with the Usearch 10 software, following the Uparse pipeline (Edgar 2013). Operational taxonomic units (OTUs) were picked at a threshold of 97% similarity and classified taxonomically by comparing representative OTU sequences with reference data from the Ribosomal Database Project (RDP version 16). Alpha diversities, sample distances and relative frequencies after dilution were calculated with the MicrobiomeAnalyst (Dhariwal et al. 2017).

### Isolation of colonic immune cell populations and analysis by flow cytometry

Mice were fed with a diet containing heme iron or iron citrate as described above. After 21 days, necropsy was performed. The entire colon was collected and rinsed with ice-cold PBS. Immune cell populations of *lamina propria* and the intraepithelial compartment were isolated as reported (Reissig et al. 2014). The immune cell populations were phenotyped using antibody staining (Supplementary Table 4) and flow cytometry with a FACSCanto™ II (BD Biosciences, New Jersey, USA). Data were processed using FlowJo software version 8.8.7 (Ashland, USA).

### Fecal water sample preparation

Fecal samples of both experimental groups were collected after 21 days and stored at − 80 °C. Sample preparation was conducted according to a protocol published (Joosen et al. 2009). Approximately 15 mg of frozen feces was thawed and diluted (0.09 g/ml) with ultrapure water (ddH2O). Samples were homogenized at 4 °C for 20 min (Retsch Mixer Mill MM400, Retsch GmbH). Afterwards samples were ultra-centrifuged (50,000*g*) at 4 °C for 2 h. The supernatant was collected immediately and stored at − 20 °C until analysis.

### Determination of fecal NOC

Fecal water samples were analyzed for apparent total nitroso compounds (ATNC) including nitrosothiols (RSNO), nitrosly iron (Fe–NO) and *N*-nitroso compounds (NOC; RNNO) as outlined in “Supplementary Methods” using a modification of the method previously described (Joosen et al. 2009).

### Ethics

All animal experiments were approved by the government of Rhineland-Palatinate and the Animal Care and Use Committee of University Medical Center, Mainz, Germany (# 23177-07/G 15-1-044). The experiments were performed in accordance with the German Federal Law and the guidelines for the protection of animals.

### Statistics

All animal experiments were performed independently at least twice, except for microbiome analyses. Depending on the endpoint (microbiome composition, tumor formation, etc.) typically 5–20 animals per group were used as indicated in the figure legends. Representative western blots and confocal microscopy images are depicted. Data were analyzed for outliers with the Grubbs' test using GraphPad Prism 8.0 software. All values are displayed as mean + standard errors of the mean (SEM). Statistical analysis was performed in GraphPad Prism 8.0 software using two-sided Student’s *t* test and statistical significance was defined as *p* < 0.05.

## Results

### Dietary heme iron triggers a persistent dysbiosis of the intestinal microbiota

First, we assessed the long-term effects on the intestinal microbiota in mice that received a diet supplemented with heme iron or an iron-balanced control diet with iron citrate (Fig. 1a). Phylogenetic analyses were conducted using next-generation sequencing of the bacterial 16S rRNA gene. Our experiments revealed that dietary heme caused a strong reduction in α-diversity already after 21 days, which persisted over the whole experiment for up to 162 days (Fig. 1b). Assessment of the ß-diversity clearly shows the formation of two different microbial clusters in response to the two diets (heme iron vs. iron citrate) (Fig. 1c). Notably, both the α- and ß-diversity were similar in the two experimental groups at the beginning of the study (Fig. 1b, c). With regard to the taxonomic composition, dietary heme induced a strong reduction in the phylum *Firmicutes* in favor of an increased number of *Proteobacteria*, which was further potentiated after 162 days (Fig. 1d). This is also reflected on the genus and family level as revealed by differential bacterial abundance analysis (Fig. S1). Within the phylum *Bacteroidetes*, both gram-negative *Bacteroidaceae* and *Prevotellacea* were significantly enriched in mice fed with heme iron (Fig. 1e). Vice versa, gram-positive *Lactobacillaceae* and *Ruminococcacea* belonging to the phylum *Firmicutes* were suppressed in mice fed with heme iron (Fig. 1f). Gram-negative *Enterobacteriaceae* from the phylum *Proteobacteria* were abundant after dietary heme iron (Fig. 1g), whereas gram-positive *Bifidobacteriaceae* were almost completely absent in this group (Fig. 1h). Taken together, dietary heme provoked a reduced α-diversity with persistent dysbiosis of the intestinal microbiota, characterized by a decrease in *Firmicutes* to *Bacteroidetes* ratio and an increase in gram-negative bacteria, particularly *Proteobacteria*.

### Heme iron induces a chronic colitis and hyperproliferation of the colon epithelium

**Fig. 2:**
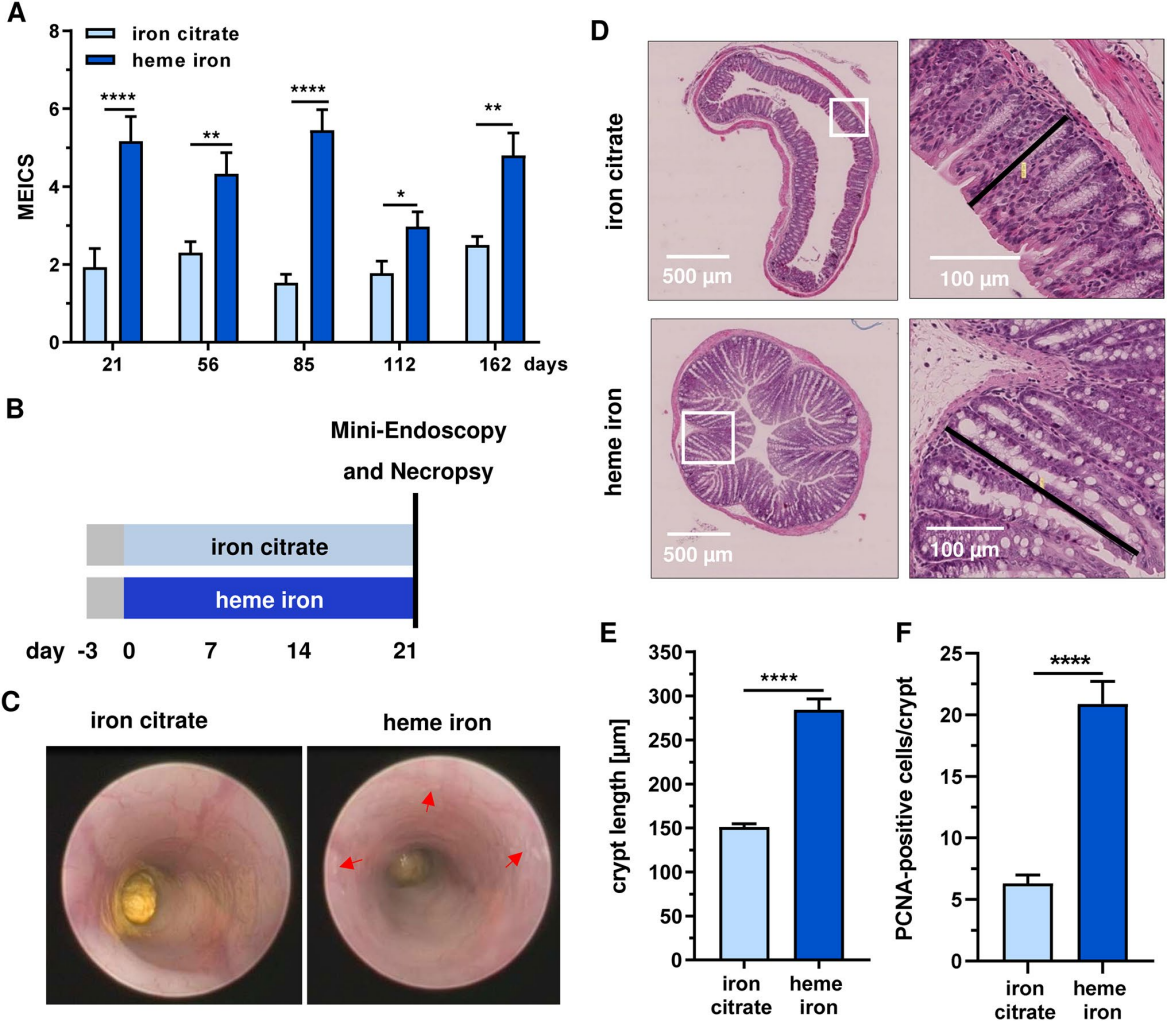
Dietary heme iron induces chronic intestinal inflammation. **a** Analysis of intestinal mucosal inflammation at indicated time points in mice upon a diet with iron citrate or heme iron. MEICS was assessed by mini-endoscopy. Data are shown as mean + SEM (*n* ≥ 10). *p < 0.05; **p < 0.01; ***p < 0.001; ****p < 0.0001. **b** Experimental set-up for short-term feeding study (21 days) **c** Representative mini-endoscopic pictures of mice in short-term study. Red arrows indicates fibrin deposition. **d** Representative images of H&E staining with magnifications (region in white box) from colorectal tissue obtained in short-term study. Black bars indicate crypt length. **e** Quantitative assessment of crypt length shown in **d**. Data are depicted as mean + SEM (*n* = 4, 6 sections per sample). ****p < 0.0001. **f** Number of PCNA-positive cells per crypt. Data are displayed as mean + SEM (*n* ≥ 5, 6–10 sections per sample). ****p < 0.0001

As intestinal dysbiosis has been linked to gut inflammation, we set out to determine the murine endoscopic index of colitis severity (MEICS) during the entire study. Intriguingly, we detected a significant increase in intestinal inflammation in mice fed with heme iron, which was evident already after 21 days and persisted until the end of the study (Figs. 2a and S3A). At the same time, the weight of the animals during the study was monitored, showing a reduced weight gain in mice receiving heme iron as compared to the iron citrate group (Fig. S2A). To detail these effects, a short-term study (21 days) was performed using the same experimental setup as before (Fig. 2b). Heme oxygenase-1 (HO-1) expression in colorectal tissue was visualized as a marker for heme iron uptake into intestinal epithelial cells, revealing a strong cytoplasmic HO-1 signal primarily observed in the upper part of the colon crypts facing the gut lumen (Figs. S2B and S2C). Importantly, ICP–MS measurements showed that the iron content in colorectal tissue was not significantly different between the two groups (*i.e.* heme iron vs. iron citrate) (Fig. S2D). Mini-endoscopy confirmed the increased colonic inflammation status in mice upon dietary heme iron, as attested by enhanced granularity and reduced translucency of the colon mucosa together with fibrin deposition (Fig. 2c). Microscopy of H&E stained colon sections showed a significant enlargement of crypt length and more mitotic figures after dietary heme (Figs. 2d, e, S3B). In agreement with these findings, the number of PCNA-positive cells per crypt reflecting the proliferative compartment increased upon heme iron intake (Fig. 2f and Fig. S3C). In summary, the intestinal dysbiosis induced by dietary heme was clearly associated with chronic gut inflammation and hyperproliferation of the intestinal epithelium.

### Dietary heme iron triggers the infiltration of myeloid immune cells into intestinal epithelium

**Fig. 3:**
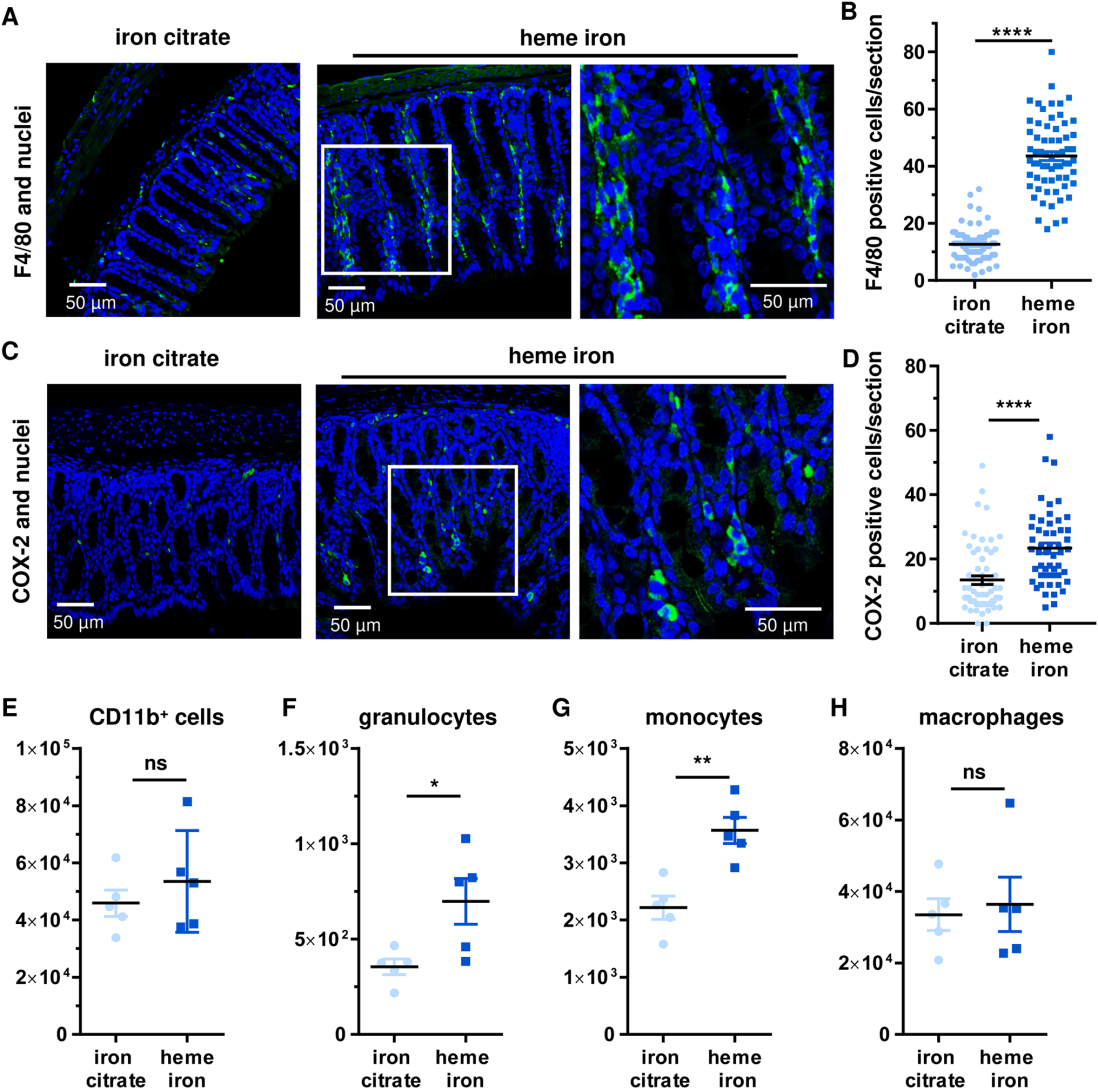
Dietary heme iron triggers the intestinal infiltration of innate immune cells. **a** Visualization of F4/80-positive cells (macrophages) in colorectal tissue obtained from mice after 21 days on a diet with heme iron or iron citrate. Shown are representative confocal microscopy images with a magnified section (white box). **b** Quantitative assessment of F4/80-positive cells per section. Data presented as mean + SEM (*n* ≥ 4, 6–10 sections per sample). ****p < 0.0001. **c** Staining of COX-2 positive cells in colorectal tissue obtained from mice as described in **a**. Shown are representative confocal microscopy images with a magnified section (white box). **d** Quantification of COX-2-positive cells per section. Data presented as mean + SEM (n ≥ 4, 6–10 sections per sample). ****p < 0.0001. **e**–**h** Flow cytometry-based analysis of myeloid immune cells in the colonic *lamina propria* obtained from mice as described in **a**. Shown is the total number of CD11b^+^ cells (e), granulocytes (**f**), monocytes (**g**) and macrophages (**h**). Data are given as mean ± SEM (*n* ≥ 4). Ns: *p* > 0.05; *p < 0.05; **p < 0.01

We then set out to analyse whether innate immune cells are activated and/or recruited to colorectal mucosa following dietary heme iron for 21 days. To this end, F4/80 positive macrophages were stained by immunohistochemistry (IHC) followed by analysis with confocal microscopy. While mice receiving the control diet displayed a low frequency, F4/80 positive macrophages were more abundant in the colorectal mucosa of heme fed animals (Fig. 3a, b). Similarly, the number of COX-2 positive cells was significantly higher in colorectal tissue of mice, which received dietary heme (Fig. 3c, d). In another set of experiments, cells of the *lamina propria* were isolated from the entire colorectum directly after necropsy and phenotyped by flow cytometry. The number of CD11b-positive myeloid cells (e.g. granulocytes, monocytes and macrophages) was elevated in the group fed with heme iron (Fig. 3e). This was largely attributable to a significant rise in granulocytes and monocytes, while the macrophage population hardly differed between the two diets (Fig. 3f–h). The latter somewhat contrasts the findings from IHC (Fig. 3a, b), but may be attributable to the different nature of the samples (entire colorectum vs. colorectal tissue section). Altogether, our findings show that dietary heme triggers an infiltration of myeloid cells into colorectal mucosa with an increased number of COX-2 positive cells, thereby promoting intestinal inflammation.

### Dietary heme affects intestinal T cell subsets and their distribution in *lamina propria* and the intraepithelial compartment

**Fig. 4:**
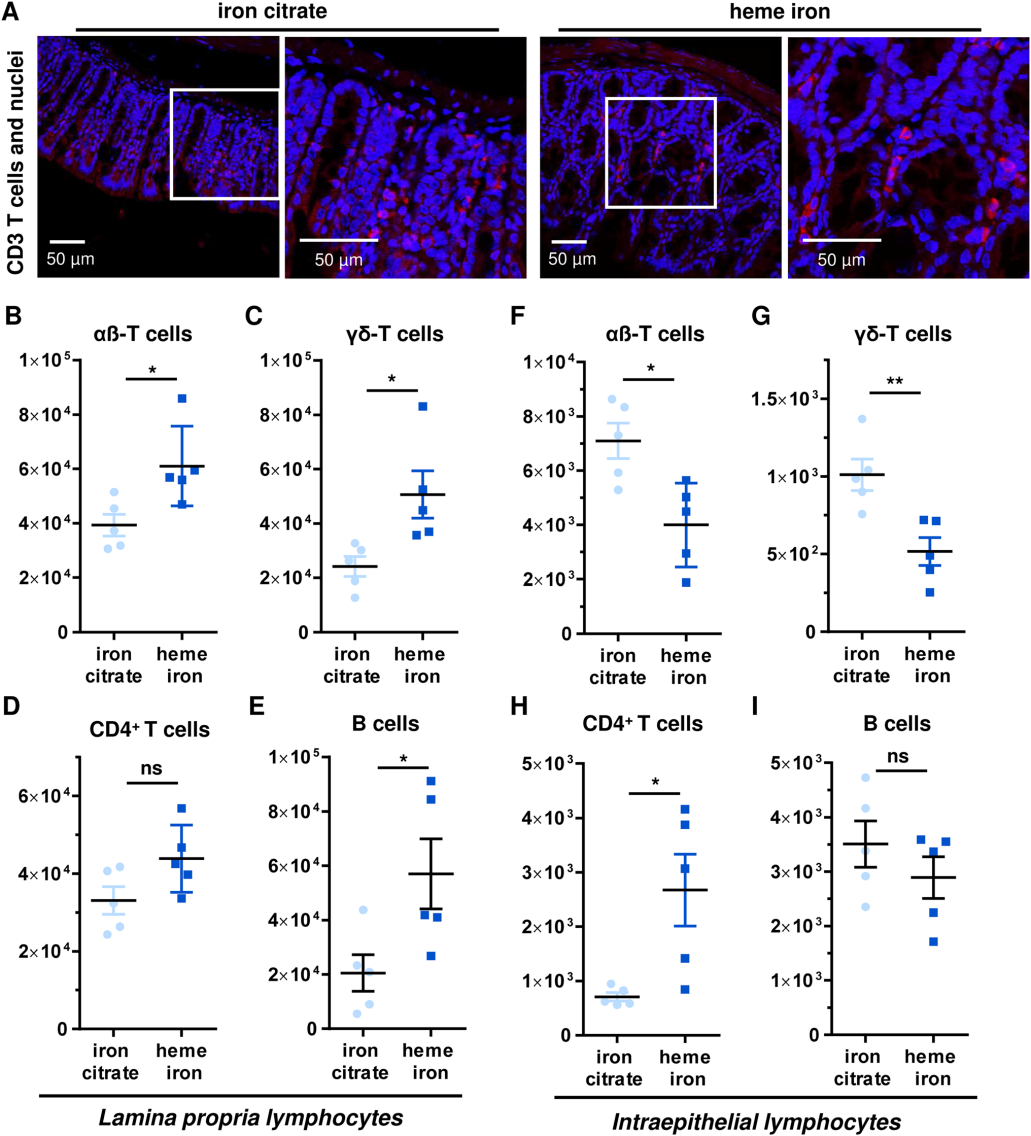
Dietary heme iron differentially affects T cell subpopulations in the *lamina propria* and the intraepithelial compartment. **a** Staining of CD3-positive T cells in colorectal tissue from mice fed with a diet containing heme iron or iron citrate for 21 days. Representative confocal images and magnified sections thereof (white box) are shown. **b**–**e** Flow cytometry-based analysis of different lymphocyte subpopulations in the *lamina propria* of mice treated as described in A. Shown is the total number of αβ-T cells (**b**), γδ-T cells (**c**), CD4^+^ T cells (**d**) and B cells (**e**). Data are depicted as mean ± SEM (*n* ≥ 4). Ns: *p* > 0.05; *p < 0.05. **f–i** Flow cytometry-based analysis of different lymphocyte subpopulations in the intraepithelial compartment of mice treated as described in A. Shown is the total number of αβ-T cells (**f**), γδ-T cells (**g**), CD4^+^ T cells (**h**) and B cells (**i**). Data are given as mean ± SEM (*n* ≥ 4). *p < 0.05; **p < 0.01; ****p < 0.0001

Next, we elucidated whether a short-term diet with heme iron (21 days) may also affect T cells and their subsets in colorectal mucosa, since these cells are important for intestinal tissue homeostasis and immune defence against luminal bacteria. At first, CD3-positive T cells were studied by immunohistochemistry and confocal microscopy. Our analysis revealed a higher number of CD3-positive T cells in colorectal tissue from mice fed with heme iron (Figs. 4a and S4A). To investigate the effects on different T cell subsets, lymphocytes were freshly isolated from both the *lamina propria* (LP) and the intraepithelial compartment (IEC) at the end of the 21-day feeding study. The T cell subpopulations were phenotyped and analyzed by flow cytometry. Dietary heme caused a significant rise of both αß-T cells and γδ-T cells in the LP as compared to the control diet with iron citrate (Fig. 4b, c). Furthermore, an elevated number of CD4-positive T helper cells was observed following dietary heme intake (Fig. 4d). Interestingly, these changes were not limited to T cells, but also observed in B cells that accumulated in the LP (Fig. 4e). At the same time, lymphocytes residing in the IEC were analyzed by flow cytometry. In contrast to the situation in the LP, mice fed with heme iron displayed a significantly reduced number of both αß-T cells and γδ-T cells as compared to mice receiving the iron-balanced control diet (Fig. 4f, g). Interestingly, more T helper cells (CD4-positive) were detected in the IEC upon dietary heme iron (Fig. 4h). Furthermore, a slight, but not significant reduction of B cells in the IEC was observed in the heme iron fed group (Fig. 4i). In conclusion, dietary heme iron was shown to increase the number of both T cells and B cells in the LP, while a significant reduction of γδ-T cells was revealed in the IEC, suggesting an impaired intestinal tissue homeostasis and immune defence at the epithelium-lumen interface.

### Dietary heme iron triggers the DNA damage response in colorectal mucosa, but attenuates intestinal epithelial apoptosis

**Fig. 5:**
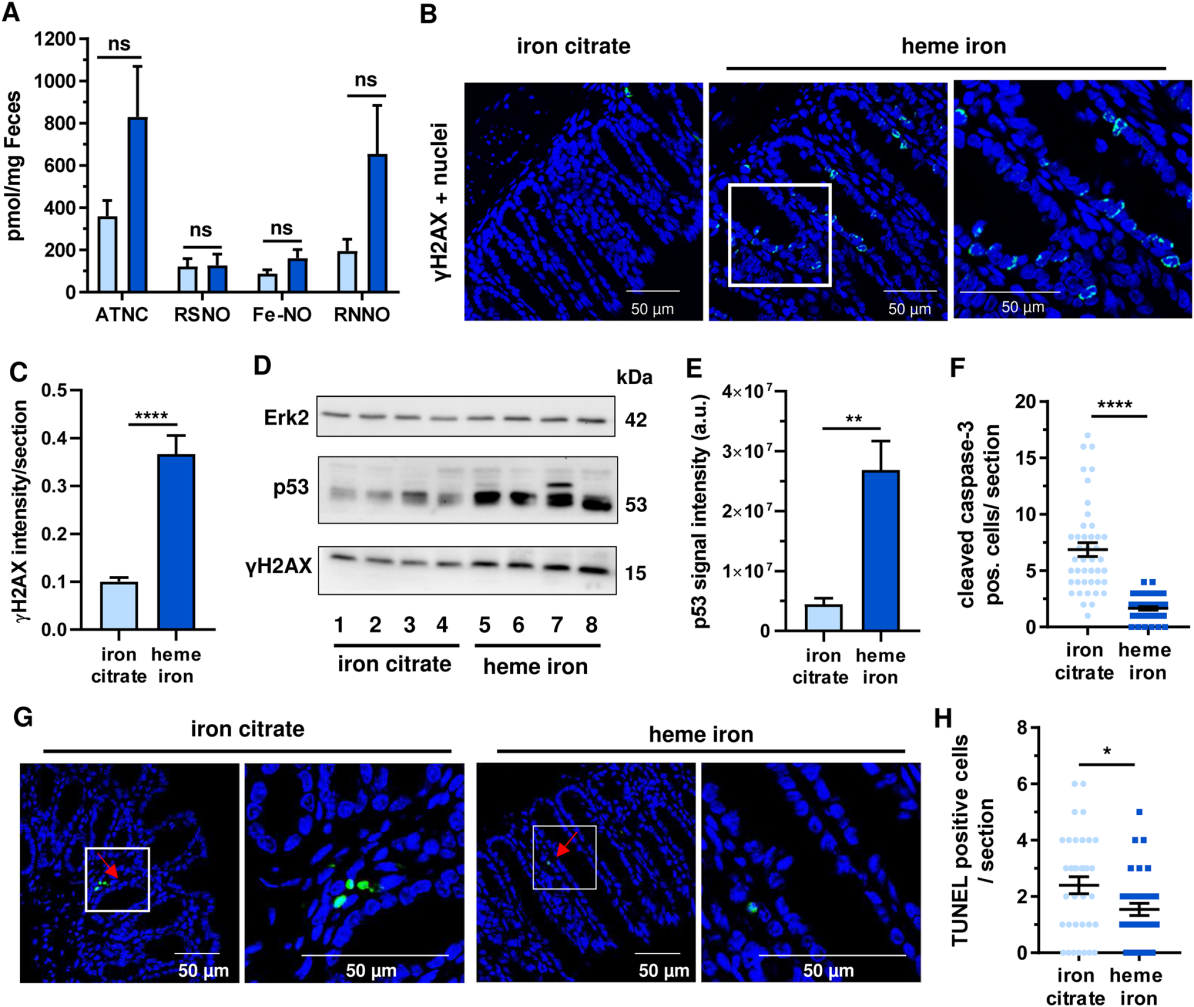
Dietary heme iron is genotoxic in colorectal tissue but impairs intestinal apoptotic cell death. **a** Analysis of fecal NOC formation determined as apparent total nitroso compounds (ATNC), comprising nitroso thiols (RSNO), nitrosyl iron (Fe–NO) and *N*-nitroso compounds (RNNO). Feces samples were obtained from mice that received a diet supplemented with heme iron or iron citrate for 21 days as described. Data are shown as mean + SEM (*n* ≥ 6). Ns: *p* > 0.05. **b** Detection of γH2AX in colorectal tissue from mice treated as described in **a**. Representative confocal images and magnified sections (white box) are displayed **c** Quantitative evaluation of γH2AX-staining. Data are depicted as mean + SEM (*n* = 4, 6–10 sections per sample). **d** Analysis of DNA damage response markers in colorectal tissue homogenates from mice treated as described above. Tissue homogenates were subjected to SDS-PAGE followed by Western blot analysis for p53 and γH2AX. Erk2 was visualized as loading control. **e** Densitometric evaluation of p53 signal intensity normalized to the loading control using ImageJ software (*n* = 4). **p < 0.01. **f** Quantitative assessment of caspase-3 cleavage in intestinal epithelium of mice treated as describe above. Data are shown as mean ± SEM (*n* = 5, 6–10 sections per sample). ****p < 0.0001. **g** Assessment of apoptotic cells in colorectal tissue by means of TUNEL staining. Representative confocal images with enlarged sections (white box) are shown. **h** Evaluation of TUNEL positive cells per section. Data are depicted as mean ± SEM (*n* ≥ 5, 6–10 sections per sample). *p < 0.05

We studied the genotoxic potential of dietary heme iron using the same experimental setup for 21 days. Initially, the formation of fecal NOC determined as apparent total nitroso compounds (ATNC) was analyzed, showing a substantially higher level of ATNC upon dietary heme (Fig. 5a). This was mainly attributable to the increased formation of *N*-nitroso compounds (here denoted as RNNO), while nitrosothiols (RSNO) and nitrosyl iron (Fe–NO) were hardly changed (Fig. 5a). As NOC are genotoxic, the formation of phosphorylated histone 2AX (γH2AX), a DNA damage marker, was investigated by IHC and confocal microscopy. Dietary heme iron significantly increased γH2AX in colorectal mucosa, which was mainly detected in the basal colon crypts (Fig. 5b, c). In contrast, the control diet with iron citrate showed only low γH2AX levels (Fig. 5b, c). Furthermore, γH2AX and the tumor suppressor p53 were studied in colorectal tissue homogenates by Western blot analysis. Consistent with the IHC results, higher levels of γH2AX were detected upon dietary heme intake (Figs. 5d and S4B). p53 was also clearly stabilized in colorectal tissue of mice fed with heme iron, highlighting its genotoxic potential (Fig. 5d, e). Since DNA damage and the resulting p53 accumulation can trigger cell death (Roos et al. 2016), we assessed caspase-3 cleavage by IHC and confocal microscopy. Contrary to our expectations, a pronounced reduction of cells positive for cleaved caspase-3 was observed in colorectal mucosa following dietary heme intake (Figs. 5f and S4C). In support of these findings, the number of TUNEL-positive cells was decreased by dietary heme (Figs. 5g, h). Taken together, our results provide evidence for genotoxicity of dietary heme in intestinal epithelial cells, which was accompanied by reduced intestinal apoptosis.

### Dietary heme iron promotes colorectal carcinogenesis in AOM-initiated mice

**Fig. 6:**
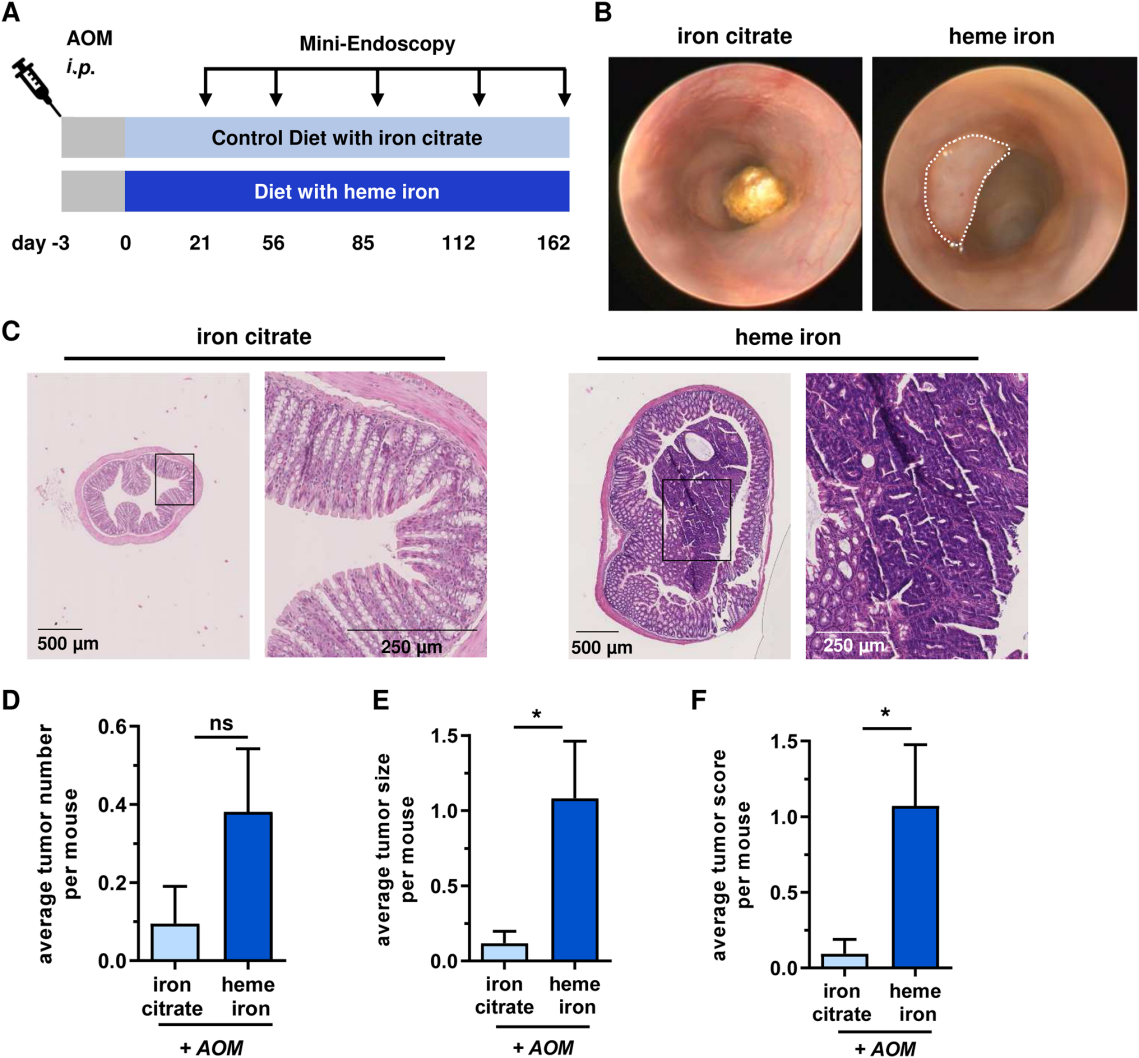
Colorectal tumor promotion by dietary heme iron. **a** Experimental schedule for tumor promoting properties of a long-term heme iron or iron citrate diet with prior *i.p*. injection with azoxymethane (AOM, 15 mg/kg bw). 72 h upon AOM injection mice were fed with iron citrate or heme iron for 162 days. To assess intestinal inflammation and tumor formation, mini-endoscopy was performed at indicated time points. **b** Representative images of mini-endoscopy at day 162. Colonic tumor in heme iron fed mice is marked with the white dotted line. **c** H&E-stained murine colon tissue according to the time point shown in B. Enlarged sections are depicted with black rectangle. **d**–**f** Statistical evaluation of mean tumor number (**d**), tumor size (**e**) and tumor score (**f**) per mouse assessed by mini-endoscopy at day 162. Data are shown as mean + SEM (*n* ≥ 21). Ns: *p* > 0.05; *p < 0.05

Finally, we studied the impact of dietary heme on colorectal carcinogenesis in a long-term experiment. To this end, mice initially received a single injection with the colonotropic tumor initiator AOM (15 mg/kg bw) followed by a diet supplemented with heme iron or iron citrate as control for 162 days (Fig. 6a). Changes in the intestinal microbiota were monitored throughout the study as described above (Fig. S5A). Consistent with the experimental setup without AOM injection (see Fig. 1), dietary heme caused a pronounced reduction in α-diversity and a persistent dysbiosis with reduced levels of *Firmicutes*, but increased levels of *Proteobacteria* (Figs. S5B-D). Gram-positive taxa (or families) such as *Lactobacillaceae* and *Bifidobacteriaceae* were suppressed following dietary heme intake, whereas gram-negative species including *Prevotellaceae* and *Enterobacteriaceae* were enriched (Fig. S5E-H). Concomitantly, intestinal inflammation was studied by non-invasive mini-endoscopy, showing significantly increased MEICS in AOM-initiated mice fed with dietary heme. The higher MEICS levels in the group with dietary heme were detected throughout the study (Figs. S6A and S6b). It should also be mentioned that heme-fed mice displayed a reduced weight gain compared to mice receiving iron citrate (Fig. S6C), which is consistent with the observed persistent inflammation. Intriguingly, colorectal tumor formation was observed in mice fed with heme iron as evidenced by mini-endoscopy and histopathology (Fig. 6b, c). AOM-initiated mice that received the control diet with iron citrate showed almost normal crypt architecture and a lower average tumor number (Fig. 6b–d). It is important to note that dietary heme intake significantly increased both the average tumor size and tumor score, a sum parameter based on both tumor number and size, as compared to dietary iron citrate (Fig. 6e, f). In conclusion, the data revealed that dietary heme promotes colorectal tumor growth, which is in line with its observed pro-inflammatory effects in the large intestine and the dysregulated intestinal microbiota.

## Discussion

Throughout this study a dose of 0.25 µmol hemin/g diet (= 163 µg hemin/g diet) was used, which was derived from a previous short-term study over 14 days with significant effects on intestinal proliferation and cytotoxicity of fecal water (IJssennagger et al. 2013). Given that C57BL/6 J mice consume 4 g food per day on average (Bachmanov et al. 2002), this corresponds to a total uptake of about 650 µg hemin per day and mouse. Red meat was reported to contain 7.1 µg heme iron/g (porc) and 12.1 µg heme iron/g (beef) (Pretorius et al. 2016). Considering the molecular weights of heme iron (56 g/mol) and hemin (652 g/mol), this translates into 82.7 µg hemin/g porc and140.9 µg hemin/g beef. According to EFSA, the average red meat consumption in Europe ranges from 26 to 87 g per day and person (Kruger and Zhou 2018). This is equivalent to an uptake of 2.15 mg–7.19 mg hemin per day using porc as main meat source. This calculation illustrates that the hemin dose used in our animal study is even below the range of the average daily human exposure across Europe. Therefore, our data underline the relevance of excessive red meat consumption in humans for colorectal carcinogenesis.

Initially, we show that dietary heme causes an intestinal dysbiosis and a reduced α-diversity of the gut microbiota, which was characterized by a decreased level of gram-positive *Firmicutes*, such as *Lachnospiraceae, Ruminococcaceae and Lactobacillaceae*. In contrast to that, gram-negative *Proteobacteria,* particularly *Enterobacteriacea*, were more abundant following dietary heme intake. The findings of our long-term study are in line with previous short-term experiments in rodents, which received higher doses of dietary heme ranging from 0.5 to 1.5 µmol/g diet (Constante et al. 2017; Ijssennagger et al. 2015; Martin et al. 2019). Former in vitro studies showed that fecal water obtained from heme-fed mice impaired the growth of *Lactobacillus plantarum* (*Firmicutes*), whereas the growth of *Escherichia coli* (*Proteobacteria*) was promoted (IJssennagger et al. 2012a). This is in line with an older report, which described an inhibiting effect of hemin on gram-positive cocci and rods at 20 µg hemin/mL (≈ 31 µM), while some gram-negative bacteria (e.g. *Bacteroides fragilis, Bilophila wadsworthia*) were not affected (Nitzan et al. 1994). Interestingly, consumption of 200 g red meat may yield luminal heme concentration of ~ 34 µM as calculated elsewhere (Kostka et al. 2020). On the other side, heme is an essential iron source required for the survival of most bacteria. Particularly pathogenic bacteria have evolved sophisticated mechanisms to sense, bind and take up heme (Huang and Wilks 2017). Both aspects likely contribute to the observed alterations in microbiota composition following dietary heme. Altogether, it is conceivable that the increased luminal availability of heme directly affects bacterial cell growth and survival.

Importantly, our study revealed that the alterations in the gut microbiota are persistent and accompanied by a steady rise in *Enterobacteriaceae*, which was also observed after initial AOM administration followed by dietary heme intake. Comparable effects were reported in IBD patients, which display a decreased diversity of the microbial community, an upregulated level of *Enterobacteriaceae* and a decreased abundance of *Firmicutes* (Kang and Martin 2017). This divergence from the normal intestinal microbiota composition was also found in humans after intake of an animal-based diet containing red and processed meat as compared to the plant-based control diet (David et al. 2014). Decreased levels of *Firmicutes* (e.g. *Eubacterium rectale, Rumimnococuccus bromii*) and increased levels of bile-tolerant bacteria, such as *Bacteroides* (*Bacteroidetes*) and *Bilophila wadsworthia* (*Proteobacteria*), were detected in this study (David et al. 2014). As noticed recently, there is still a knowledge gap regarding red meat consumption in humans and its impact on the intestinal microbiota (Albracht-Schulte et al. 2021). Therefore, further human intervention studies are required to detail the role of red meat and heme iron on the intestinal microbiota.

Intestinal dysbiosis has been linked to an increased permeability of the gut barrier (Kang and Martin 2017). Interestingly, we observed a higher abundance of *Prevotellaceae* upon dietary heme intake. *Prevotella* were shown to produce mucin-desulfating sulfatases involved in mucin degradation (Wright et al. 2000). Furthermore, dietary heme was reported to promote the abundance of mucin-degrading bacteria such as *Akkermansia muciniphila* and to reduce Muc1 expression in mucosal cells (Ijssennagger et al. 2013; 2015). Muc1 is a membrane-bound glycoprotein, which forms the mucus barrier together with secreted Muc2 as major constituent (Johansson et al. 2011). In support of these findings, rats fed with heme iron displayed a higher mucosal permeability as revealed by administration of ^51^Cr-EDTA (Martin et al. 2019). Nevertheless, more studies are required to elucidate how dietary heme affects the mucus barrier and to what extent a deregulated intestinal microbiota is involved in this process.

Data from both mouse models and humans show a tight link between a compromised gut barrier and intestinal dysbiosis on the one hand, and chronic intestinal inflammation on the other hand (Neurath 2019). Here, we were able to demonstrate for the first time that dietary heme crucially affects these key pathways. It causes intestinal inflammation, which persisted over the whole study as shown by regular non-invasive mini-endoscopy. In line with this observation, an infiltration of myeloid immune cells (granulocytes, monocytes, macrophages) into the LP and an increased number of COX-2 positive cells were detected in the colorectal mucosa of mice after dietary heme intake for 21 days. COX-2 catalyzes the synthesis of pro-inflammatory mediators such as PGE2 and contributes to CRC progression (Markowitz and Bertagnolli 2009). Myeloid cells recruited to the site of inflammation and cytokine release are known to increase proliferation of intestinal epithelial cells in order to promote tissue repair (Thoo et al. 2019). In this regard, it was reported that a dysfunctional barrier triggers ß-catenin signaling via infiltrated myeloid cells to promote tissue repair in the lung (Zemans et al. 2011). Indeed, a stimulation of the entire WNT signaling pathway and an increased proliferation of intestinal epithelium by dietary heme iron were reported previously (de Vogel et al. 2008; IJssennagger et al. 2012b). In support of these findings, our histopathological analysis revealed a pronounced enlargement of colon crypts with increased proliferation of intestinal epithelial cells as attested by PCNA staining and increased mitotic index.

Furthermore, we observed an increased number of CD3-positive T cells in the colorectal mucosa. A more detailed analysis of the T cell subsets revealed a higher number of both αß- and γδ-T cells in the LP of mice fed with heme iron. Accordingly, higher levels of T helper (Th) cells were found in the LP. In stark contrast, both intraepithelial αß- and γδ-T cells declined following dietary heme, whereas intraepithelial Th cells were found enriched. Intraepithelial γδ-T cells are crucial for mucosal tissue homeostasis and are known to limit the translocation of intestinal bacteria across the gut barrier (Nielsen et al. 2017). Their protective function against intestinal inflammation is highlighted by the fact that mice deficient for γδ-T cells are prone to both chemically induced colitis and age-associated spontaneous colitis (Inagaki-Ohara et al. 2004). Furthermore, mice lacking γδ-T cells displayed an increased formation of AOM-induced colorectal tumors (Matsuda et al. 2001). Together with these studies, the depletion of γδ-T cells following dietary heme intake is likely involved in the observed chronic inflammatory and tumor promoting effects. Regulatory T cells are also relevant in this context and, therefore, deserve a closer attention in upcoming studies.

Our work is the first to show that dietary heme at low doses (0.25 µmol/g diet) increases the formation of fecal ATNC, particularly the fraction of genotoxic NOC, which was seen already after 21 days. This is in agreement with a previous study in rats fed with 1% hemoglobin (0.6 µmol heme/g diet) for 100 days, in which an increased ATNC level was detected (Bastide et al. 2015). A higher level of fecal ATNC was also found in a human randomized crossover study following red meat intake (Joosen et al. 2009). NOC are important in the etiology of colorectal carcinogenesis, since they cause DNA alkylation damage including *O*^6^-methylguanine (Fahrer and Kaina 2013). This DNA lesion can give rise to G → A transition mutations in *KRAS* as detected in human CRC. Interestingly, two previous studies indicated that red meat consumption could increase *O*^6^-MeG levels (Le Leu et al. 2015; Winter et al. 2011).

In line with the increased fecal NOC levels detected in our study, we provided evidence that dietary heme iron is genotoxic in intestinal epithelium as reflected by an increased formation of γH2AX and p53 accumulation, which are established genotoxicity markers in the colon (Fahrer et al. 2015). The findings are supported by a previous study using high levels of dietary heme (1.5 µmol/g diet) for 46 days (Bastide et al. 2015). The observed γH2AX formation is likely attributable to replication blocking DNA lesions and/or DNA strand break induction induced by genotoxic NOC. Consistent with this notion, a higher level of DNA strand breaks was detected by the alkaline Comet assay in mucosal cells, which were isolated from rats fed with heme (1.5 µmol/g diet) (Martin et al. 2019). Interestingly, DNA strand break induction by heme iron was also observed in primary-like human colonocytes (Seiwert et al. 2020b). Whether this occurs directly or because of the induction of oxidative DNA lesions and DNA alkylation damage warrants further investigation.

Since apoptotic cell death is an important barrier in carcinogenesis by eliminating pre-neoplastic cells, we analyzed apoptosis induction in colorectal tissue. Our findings showed a significantly reduced number of cells positive for cleaved caspase-3 as well as a decrease in TUNEL-positive cells in the colorectal mucosa of mice fed with heme iron, indicative of reduced apoptosis levels. This is in agreement with two short-term studies with higher heme levels (0.5 µmol/g diet), in which a reduced caspase-3 activity in mucosal homogenates and an upregulation of the anti-apoptotic NF-κB target genes *survivin/birc5* and *xiap* in the colon mucosa were reported (de Vogel et al. 2008; IJssennagger et al. 2012b). A reduced level of apoptosis in intestinal epithelium despite a high level of DNA alkylation damage has been shown to favor the outgrowth of preneoplastic cells and tumor induction using DNA repair defective mouse models (Dörsam et al. 2018). Furthermore, non-malignant human colonocytes have been reported to be more sensitive towards heme iron than malignant CRC cell lines (Seiwert et al. 2020b). Taken together, it is reasonable to conclude that the increased DNA damage and reduced apoptosis levels contribute to the tumor-promoting activity of dietary heme.

Here, we also demonstrated for the first time that chronic uptake of dietary heme at a low dose (0.25 µmol/g diet) is sufficient to promote colorectal tumor formation in mice after a single treatment with the colonotropic tumor initiator AOM. This extends the findings of previous studies showing that dietary heme or hemoglobin at high doses (≥ 1 µmol/g diet) increases the number of pre-neoplastic lesions such as aberrant crypt foci (Pierre et al. 2003) and elevates tumor load in the small intestine of APC^Min/+^ mice (Bastide et al. 2015), a genetic model for intestinal tumorigenesis. Up to now, there is no evidence that dietary heme is capable of inducing CRC in the absence of initiating events such as DNA alkylation damage or without genetic predisposition. However, it should be kept in mind that genetic and epigenetic inactivation of DNA mismatch repair genes and the base excision repair gene *MYH* involved in the removal of oxidative DNA damage are found in hereditary and sporadic CRC (Markowitz and Bertagnolli 2009; Vodicka et al. 2020). The consumption of high amounts of heme iron from red meat might further increase the risk for developing CRC in individuals exhibiting this DNA repair deficient phenotype. It is also noteworthy that about 40% of all patients with sporadic CRC display epigenetic inactivation of the DNA repair gene *MGMT* in colorectal tumors and precursor lesions (Fahrer and Kaina 2013). MGMT inactivation predisposes to alkylation-induced DNA damage, which is associated with G → A transition mutations in the *KRAS* oncogene, thus driving tumor growth (Fahrer and Kaina 2013). Intriguingly, a large cohort study revealed an association between dietary heme intake and an increased risk of CRC, associated with G → A transition mutations in *KRAS* and *APC* (Gilsing et al. 2013).

In summary, our study demonstrates that dietary heme causes intestinal dysbiosis and chronic intestinal inflammation, which is mediated by infiltrated myeloid cells and a redistribution of T cell subsets within the LP and the IEC. Furthermore, the data show that dietary heme causes genotoxic effects in the intestinal epithelium and, at the same time, suppresses apoptosis. These complex heme-triggered responses in intestinal epithelial cells and immune cells in the gut are crucial for driving colorectal tumor growth.

## Supplementary Information

Below is the link to the electronic supplementary material.

Supplementary file1 (DOCX 5046 kb)
