# Supplementary material for: Chronic intestinal inflammation drives colorectal tumor formation triggered by dietary heme iron in vivo
Source: Arch Toxicol. 2021 May 12;95(7):2507–22. doi: 10.1007/s00204-021-03064-6 (PMC8241717; doi:10.1007/s00204-021-03064-6)
Supplement: Supplementary file 1 — Supplementary file1 (DOCX 5046 kb) [file 204_2021_3064_MOESM1_ESM.docx]

**Supplementary Methods**

**Determination of fecal NOC**

Fecal water samples were analyzed for apparent total nitroso compounds (ATNC) including nitrosothiols (RSNO), nitrosly iron (Fe-NO) and *N*-nitroso compounds (NOC; RNNO) using an Ecomedics CLD 88 Exhalyzer (Eco Physics GmbH, Hürth, Germany). Each fecal water sample was aliquoted into three parts. One aliquot was pretreated with sulfanilamide (30 mg/ml in 1 M HCl for 5 min) to remove nitrite. This sample was used for the determination of ATNCs. The second aliquot was pretreated with aqueous HgCl_2_ (14,5 mg/ml in _dd_H_2_O) for 30 min to obtain mercury (II) stable nitroso compounds (RSNO). After 30 min of pretreatment, nitrite was removed with sulfanilamide. To obtain potassium ferricyanide stable nitroso compounds (Fe-NO) the third sample was pretreated with a K_3_Fe(CN)-solution (38 mg/ml in _dd_H_2_O for 30 min) followed by sulfanilamide treatment. All samples were stored and pretreated in the dark on ice. Analysis was also conducted in the dark to exclude any influence of light. 100 µl of each pretreated fecal water sample was injected into a purge-vessel containing 8 ml glacial acetic acid, 2 ml aqueous potassium iodide (50 mg/ml), 0.4 ml aqueous cupric sulfate (50 mg/ml) and 100 µl antifoam, kept at 3 °C. Nitrogen was bubbled through a glass frit to mix the sample and transfer released NO to the Ecomedics CLD 88 analyzer via a condenser, a NaOH trap (1 mol/l, 4 °C) and a polypropylene filter (0.2 µm, Whatmann, USA). The chemiluminescence signal resulting from the reaction of ozone and NO was detected and processed using the instrument software. The quantification was conducted by preparing a standard curve of known sodium nitrite standards in ultrapure water (3 - 100 nM). The area under curve of each signal obtained after injection of the fecal water samples was determined and compared to the area under curve of the known sodium nitrite standards. Nitrosothiols (RSNOs) were determined as the difference between mercury (II) stable and unstable compounds, Fe-NO as the difference between ferricyanide stable and unstable compounds and RNNOs as the difference between ATNCs and the sum of RSNOs and Fe-NO. Results are expressed as pmol of NO compound per milligram fecal sample.

**Supplementary Figures**

**Figure S1**

**
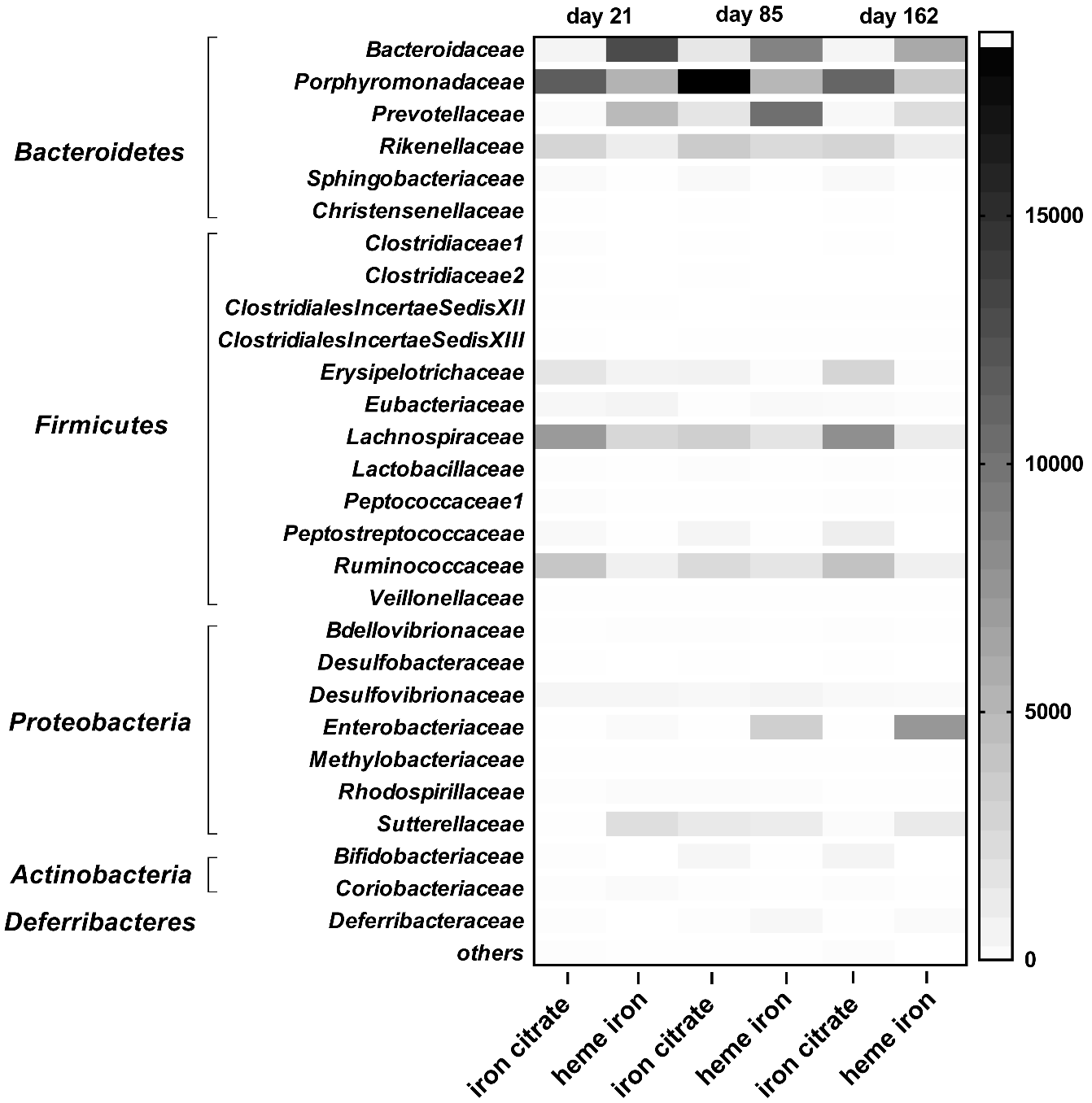
**

**Figure S1: Differential bacterial abundance analysis and impact of dietary heme iron on intestinal microbiome.** Mice received a diet containing heme iron or iron citrate for up to 162 days. Fecal samples were collected freshly after 21, 85 and 162 days. Bacterial DNA was isolated and used for next generation sequencing of the 16s rRNA gene. Data given as mean of the total bacteria number are illustrated as heat map (n=5 per group and time point).

**Figure S2**

**
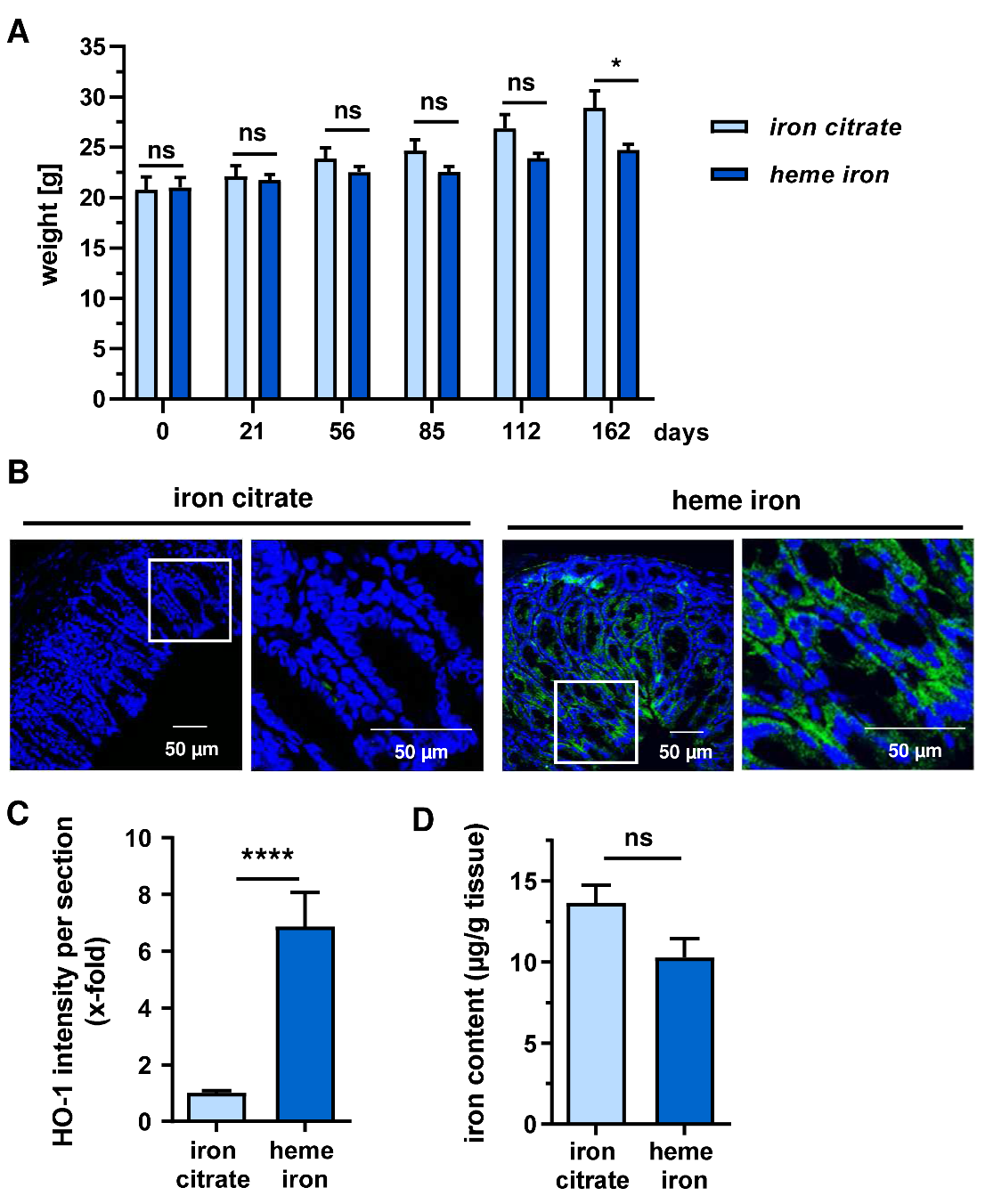
**

**Figure S2: Weight gain, iron levels in colorectal tissue and intestinal HO-1 expression.** **A** Mice received a diet containing heme iron or an iron balanced control diet with iron citrate for up to 162 days. Murine weights were monitored as indicated. Data are given as mean + SEM (n≥11 per group). Ns, p>0.05, *p<0.05. **B** Mice were fed with a diet containing heme iron and iron citrate, respectively, for 21 days. Representative confocal images of heme oxygenase-1 (HO-1) staining in colorectal tissue. Enlarged sections are indicated by a white box. **C** Quantitative evaluation of HO-1 staining. Data represents mean + SEM (n≥5 per group, 8-10 sections per sample). ****p<0.0001. **D** Iron levels in colorectal tissue obtained from mice treated as described in A. Iron content was determined by ICP-MS analysis. Data are shown as mean + SEM (n=6 per group). Ns: p>0.05.

**Figure S3**

**
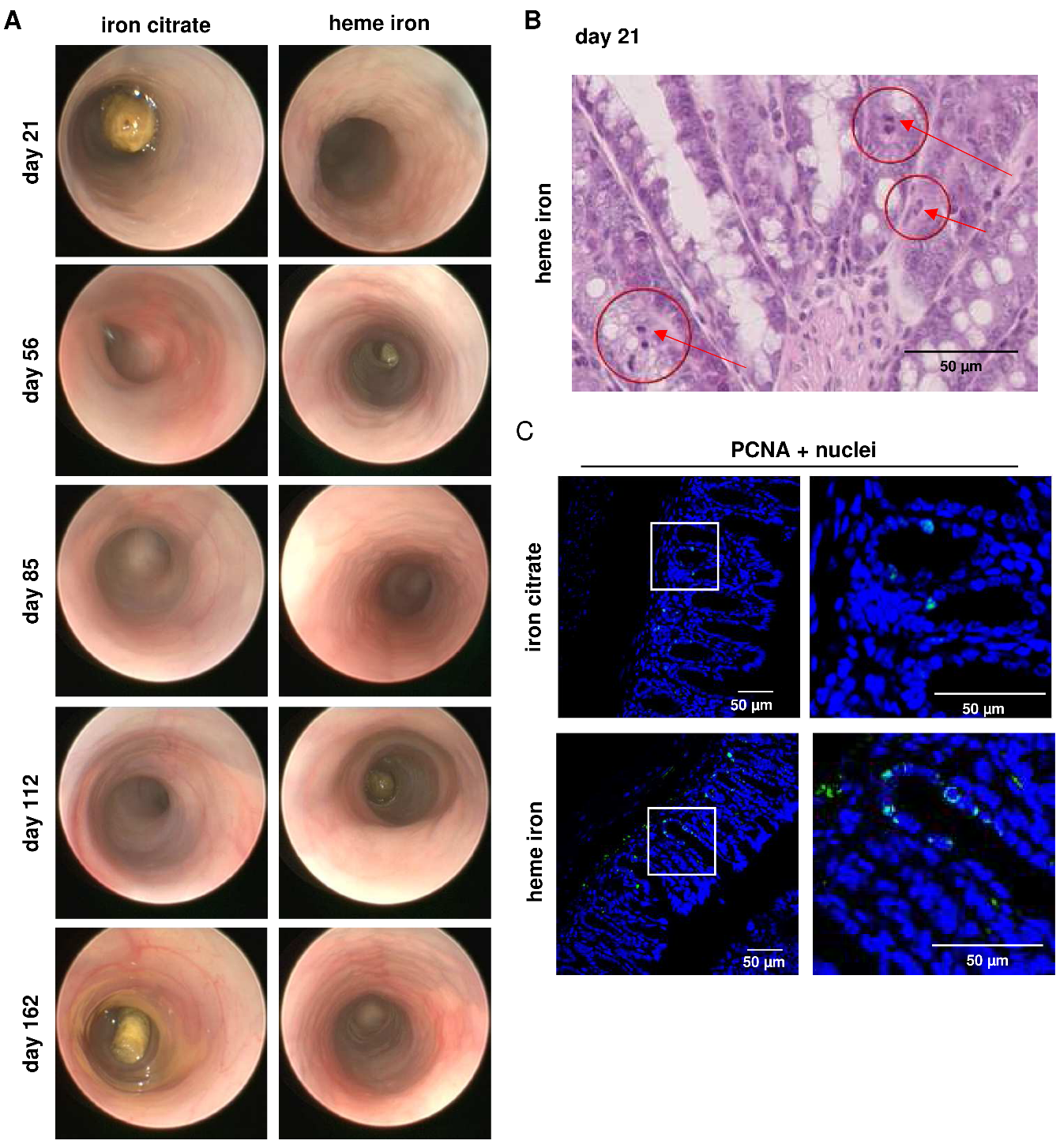
Figure S3: Dietary heme iron triggers chronic intestinal inflammation and promotes proliferation of intestinal epithelial cells.** **A** Time-dependent analysis of intestinal inflammation by non-invasive mini-endoscopy in mice upon a diet with iron citrate or heme iron. Representative pictures are displayed. **B** Mitotic figures (indicated as red arrows) in H&E stained colorectal tissue section of a heme-fed mouse after 21 days of diet. **C** PCNA staining of colorectal tissue obtained from mice following a diet with heme iron or iron citrate for 21 days. Enlarged sections are indicated with a white box. Representative confocal images are shown.

**Figure S4**

**
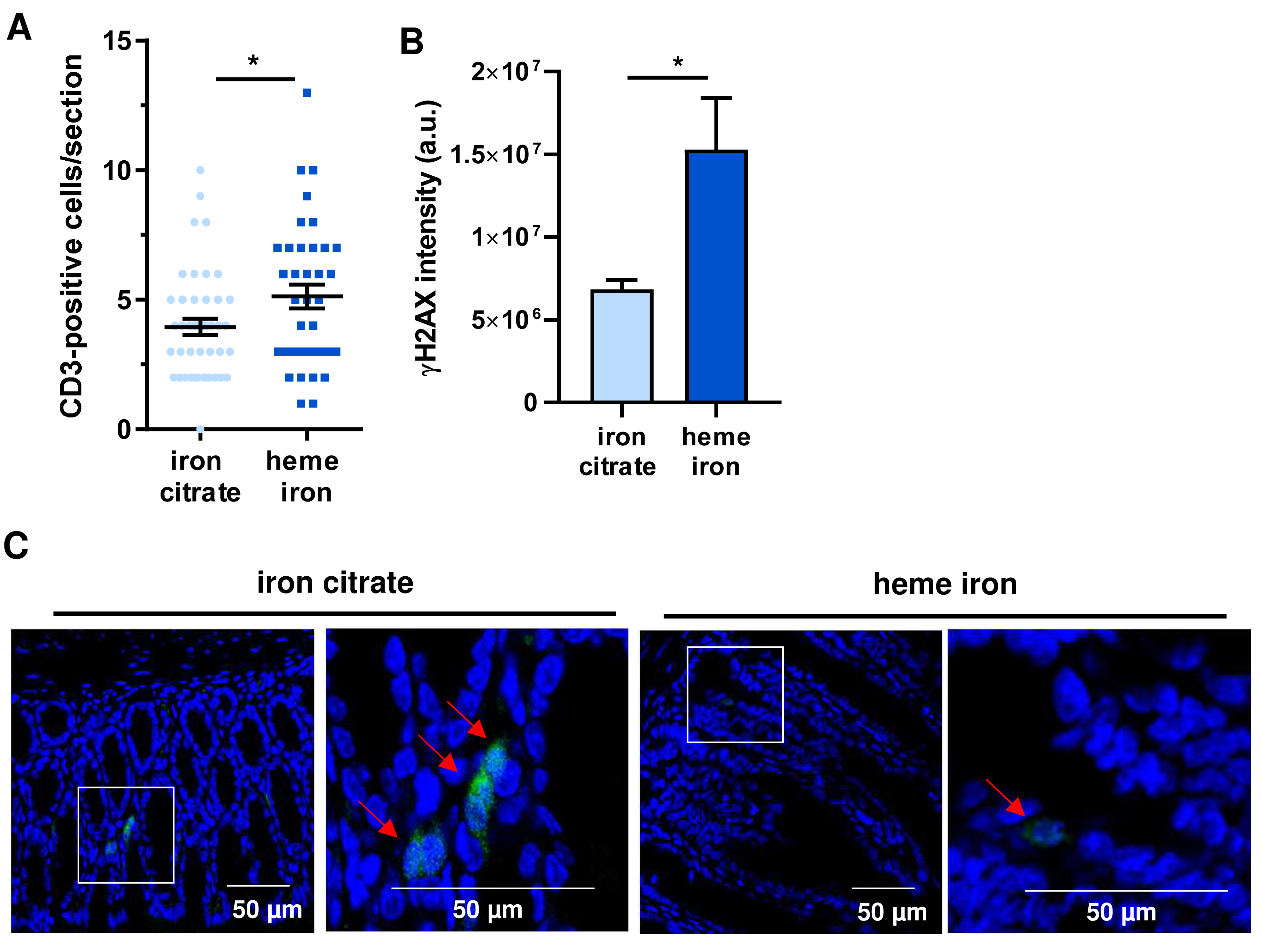
Figure S4: Effects of dietary heme iron on lymphocytes, DNA damage and apoptosis in colorectal mucosa. A** Staining of CD3-positive T cells in colorectal tissue from mice fed with a diet containing iron citrate or heme iron for 21 days. Data are given as mean ± SEM (n≥5 per group, 6-10 sections per sample). *p<0.05. **B** Western blot analysis of γH2AX as DNA damage marker in colorectal tissue homogenates from mice that received a diet supplemented with heme iron or iron citrate as described in A. Densitometric evaluation of γH2AX signal intensity normalized to the loading control Erk2 using ImageJ software (n=4). *p<0.05. **C** Detection of cells positive for cleaved caspase-3 in colorectal mucosa from mice treated as described in A. Representative confocal images and magnified sections (white box) are shown. Cleaved caspase-3 positive cells are indicated by red arrows.

**Figure S5**

**
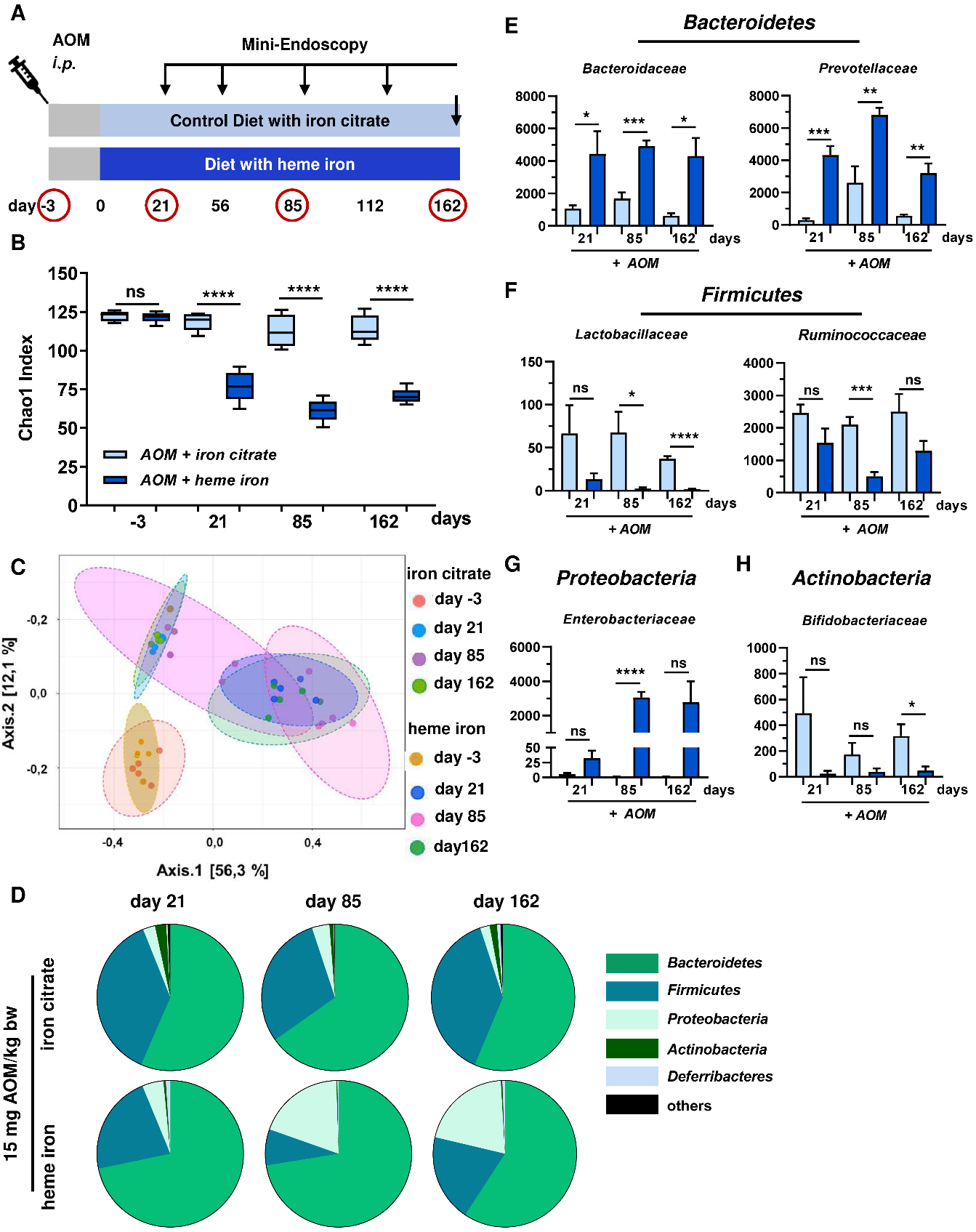
**

**Figure S5: Dietary heme causes persistent intestinal dysbiosis with reduced α-diversity in AOM-initiated mice.** **A** Experimental setup. C57BL/6 mice received an initial *i.p*. injection of AOM (15 mg/kg bw) (day -3). After 72 h, the mice were set on a diet supplemented with heme iron or a control diet with iron citrate for up to 162 days. Mini-endoscopy was performed at indicated time points. Fecal samples for microbiome analysis were freshly collected at the indicated time points (red circles). **B** α-diversity of the intestinal microbiome shown as Chao1 Index. Data are depicted as median with minimum to maximum (n=5 mice per group and time point). Ns: p>0.05; ****p<0.0001. **C** β-diversity of the intestinal microbiome shown as Bray Curtis dissimilarity. Shown are single data points. **D** Mean distribution of selected intestinal bacterial phyla in mice after 21, 85 and 162 days diet with an initial AOM injection (n=5 per group and time point). **E - H** Relative abundance of *Bacteroidetes* (E), *Firmicutes* (F), Proteobacteria (G), and *Actinobacteria* (H) after 21, 85 and 162 days iron citrate or heme iron diet with an initial AOM injection. Shown are mean + SEM (n=5). Ns: p>0.05; *p<0.05; **p<0.01; ***p<0.001; ****p<0.0001.

**Figure S6**

**
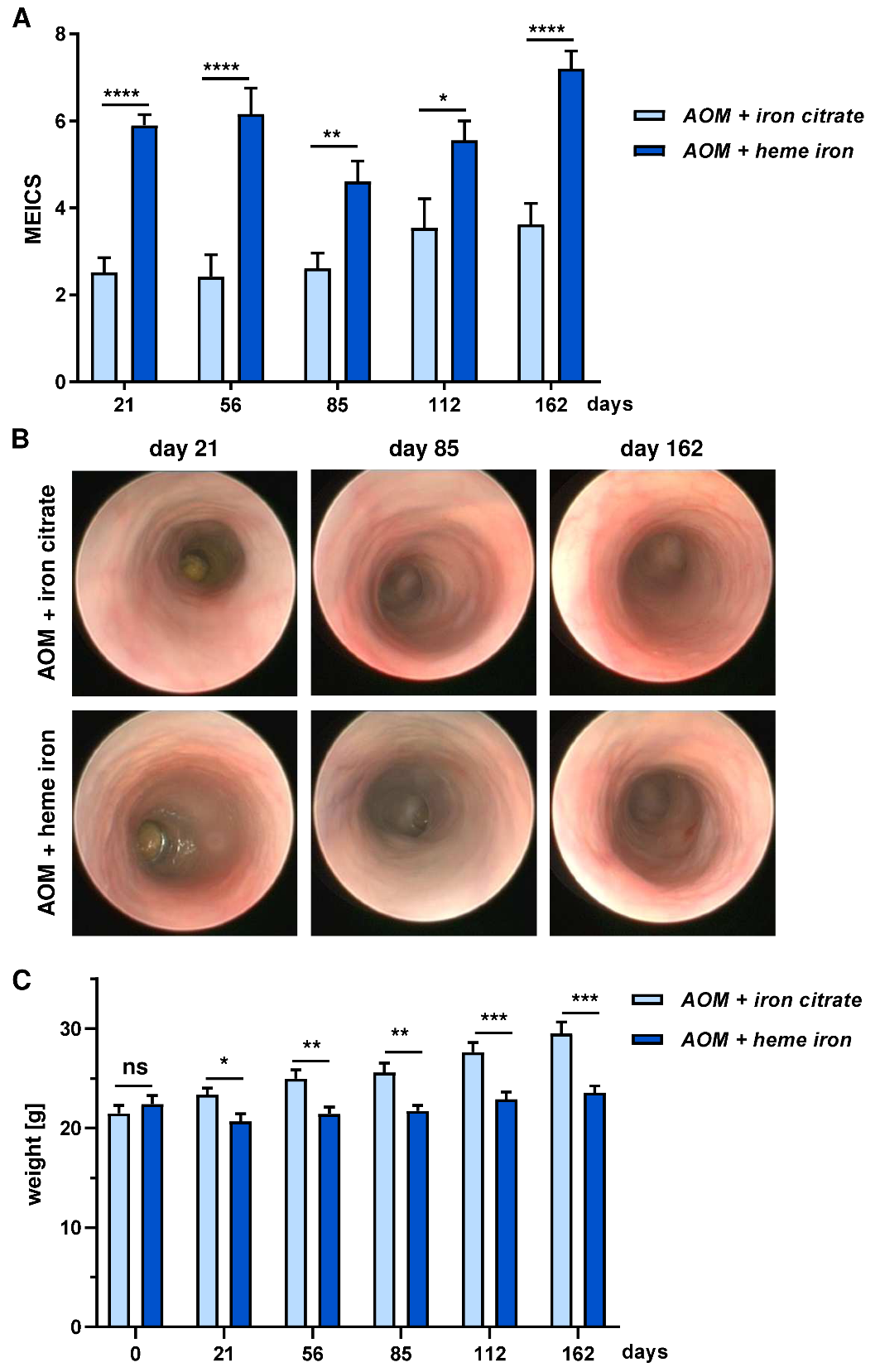
Figure S6: Dietary heme iron causes a persistent gut inflammation in AOM-initiated mice. A** Mice received an initial *i.p*. injection with the alkylating agent and tumor initiator AOM (15 mg/kg bw) followed by a diet containing heme iron or iron citrate for 162 days. MEICS was assessed by mini-endoscopy at the indicated time points. Data are shown as mean + SEM (n≥18). *p<0.05; **p<0.01; ****p<0.0001. **B** Representative mini-endoscopic images. **C** Murine weights determined as indicated. Data are given as mean + SEM (n≥18). Ns p>0.05; *p<0.05; **p<0.01; ***p<0.001.

**Supplementary Tables**

| **Ingredient** | **Unit** | **Control diet with inorganic iron supplemented as ferric citrate** | **Diet with heme iron supplemented as hemin** |
| --- | --- | --- | --- |
| Crude protein | mg/kg | 174534.071 | 174527.716 |
| Crude fat | mg/kg | 50513.814 | 50512.543 |
| Crude fibre | mg/kg | 30021.443 | 30017.63 |
| Crude ash | mg/kg | 46762.735 | 46683.454 |
| Moisture | mg/kg | 57498.42 | 57394.91 |
| Disaccharides | mg/kg | 434360.5 | 435526.7 |
| Polysaccharides | mg/kg | 190294.613 | 189163.423 |
| Calcium | mg/kg | 800.717 | 800.049 |
| Iron | mg/kg | 17.261 | 17.215 |

**Table S1: Composition of experimental diets**

**Table S2: Antibodies used for SDS-PAGE and Western blot analysis**

| **Antibody** | **Catalog No.** | **Provider** |
| --- | --- | --- |
| Anti-p53 (1C12), monoclonal mouse | 2524 | Cell Signaling Technology,  Danvers, Massachusetts, USA |
| Anti-Erk2, monoclonal mouse | sc-1647 | Santa Cruz Biotechnology,  Heidelberg, Germany |
| Anti-γH2AX, monyclonal rabbit | ab11174 | Abcam, Cambridge, UK |
| Goat-anti-Mouse -HRP  (IgGκ binding protein) | sc-516102 | Santa Cruz Biotechnology,  Heidelberg, Germany |
| Goat-anti-Mouse -HRP (IgG) | sc-2005 | Santa Cruz Biotechnology,  Heidelberg, Deutschland |
| Goat-anti-Rabbit-HRP | sc-2004 | Santa Cruz Biotechnology, Heidelberg, Germany |

**Table S3: Antibodies used for IHC and confocal microscopy**

| **Antibody** | **Catalog No.** | **Provider** |
| --- | --- | --- |
| Anti-CD3, monoclonal rat | MCA500A488 | AbD Serotec MCA, Kidlington, UK |
| Anti-cleaved caspase 3 (Asp175), polyclonal rabbit | #9661 | Cell Signaling Technology,  Danvers, Massachusetts, USA |
| Anti-COX-2, monoclonal mouse | 610204 | BD Transduction Laboratories,   Franklin Lakes, USA |
| Anti-F4/80, monoclonal rat | BM4007 | OriGene Europe, Acris Antibodies, Herford, Germany |
| Anti-HO-1, polyclonal rabbit | GTX101147 | GeneTex, Irvine, California, USA |
| Anti-PCNA, monoclonal mouse | sc-56 | Santa Cruz Biotechnology,  Heidelberg, Germany |
| Anti-γH2AX, monoclonal rabbit | ab11174 | Abcam, Cambridge, UK |

**Table S4: Antibodies used for phenotyping of immune cells by flow cytometry**

| **Antibody** | **Dilution** | **Catalog No.** | **Cell population** | **Provider** |
| --- | --- | --- | --- | --- |
| Anti-CD11b-APC/Cy7 | 1:200 | 101226 | Monocyten, Macrophages, Granulocytes | BioLegend, San Diego, California, USA |
| Anti-CD45-V510 | 1:200 | 103138 | Leukocytes | BioLegend, San Diego, California, USA |
| Anti-CD4-APC | 1:200 | 100412 | CD4 T cells | BioLegend, San Diego, California, USA |
| Anti-F4/80 APC | 1:75 | 123116 | Macrophages | BioLegend, San Diego, California, USA |
| Anti-Gr1 V450 | 1:100 | 560454  560453 | neutrophile Granulocytes | Becton Dickinson, New Jersey, USA |
| Anti-Ly-6G-PE | 1:200  SA-PE 1:200 | 127608 | neutrophile Granulocytes | BioLegend, San Diego, California, USA |
| Anti-mouse-CD45R /B220-PerCP | 1:200 | 103234 | B cells | BioLegend, San Diego, California, USA |
| Anti-TCRβ-Biotin | SA-PE/Cy7 1:100 | 553169 | αβ T cells | Becton Dickinson, New Jersey, USA |
| Anti-TCRγδ-PE | 1:200 | 12-5711 | γδ T cells | Thermo Fisher Scientific, Braunschweig, Germany |
| Streptavidin-PE (SA-PE) | 1:200 | 554061 |  | Becton Dickinson, New Jersey, USA |
| Streptavidin-PE/Cy7 (SA-PE/Cy7) | 1:200 | 25-4317 |  | Thermo Fisher Scientific, Braunschweig, Germany |
| Viab-APC/Cy7 | 1:1000 | 65-0865 | Viability marker | Thermo Fisher Scientific, Braunschweig, Germany |
